# Telomere Attrition and p53 Response 1 (TAPR1): a new player in cancer biology?

**DOI:** 10.6061/clinics/2021/e2997

**Published:** 2021-05-27

**Authors:** Gabriel Arantes dos Santos, Sabrina T. Reis, Katia Ramos Moreira Leite, Miguel Srougi

**Affiliations:** I Laboratorio de Investigacao Medica (LIM55), Departamento de Urologia, Faculdade de Medicina FMUSP, Universidade de Sao Paulo, Sao Paulo, SP, BR.; II Instituto D'Or de Pesquisa e Ensino (IDOR), Sao Paulo, SP, BR.; III Centro Universitario Atenas (UniAtenas), Passos, MG, BR.; IV Universidade do Estado de Minas Gerais (UEMG), Passos, MG, BR.

Dear editor,

Telomeres are the physical ends of eukaryotic chromosomes, and their structures are essential to maintain genome stability and limit cell proliferation (1). Normally, the replication of telomeric DNA is done by a molecule called telomerase, which is an enzyme that is inactive in the vast majority of somatic cells. Therefore, every time our cells go into mitosis, we lose pieces of the telomeres (2). When telomeres become severely short (as a natural consequence of aging or due genotoxic stress), a cellular response is triggered by p53, which causes the cell to become senescent or induces apoptosis (3).

Cancer cells must avoid the regulatory role of the telomere-p53 axis to ensure replicative immortality. To achieve this, they need to activate a telomere-maintenance mechanism (TMM), in which 90% of the cells reactivate telomerase, and 10% activate alternative lengthening of telomeres (ALT) (4,5). Another important point is that cells with deficiencies in p53 continue to multiply even with critically short telomeres, which promotes severe genetic instability (6).

Both of these phenomena highlight the central role of the telomere-p53 axis in the process of carcinogenesis. Recently, Benslimane et al. uncovered the function of a previously unannotated gene, *C16ORF72*, which they renamed Telomere Attrition and p53 Response 1 (*TAPR1*) (7). Using genome-wide CRISPR screening, they found that *TAPR1* is a novel p53 regulator that protects the cell against telomerase inhibition (and therefore, telomere shortening), as well as p53-induced senescence/apoptosis. This placed this newly identified protein in a key position at the nexus of telomere integrity and p53 regulation.

Considering the functions of *TAPR1*, the authors suggest that this molecule may have a role in cancer biology by suppressing p53 activity. Therefore, we tried to gather evidence for this by performing an analysis of this gene in the 33 tumors of The Cancer Genome Atlas (TCGA) using cBioPortal, GEPIA, and UALCAN (8-11).

First, we show the somatic alteration landscape of *TAPR1* across TCGA cancers (Figure 1A). Proportionally, the cancers with more changes in *TAPR1* are bladder urothelial carcinoma, mature B-cell neoplasm, and invasive breast carcinoma. Despite this, genetic alterations in *TAPR1* are not common (especially mutations) and are absent in 10 out of 33 types of cancer. Additionally, alterations in *TAPR1* do not change the overall and disease-free survival of patients (data not shown).

The authors who identified this gene also reported a genetic interaction between *TAPR1* and TERT (telomerase protein subunit), ACD (or TPP1, a protein involved in recruiting telomerase), and TP53. Interestingly, we noticed a trend of co-occurrence between mutations in *TAPR1* with TERT (odds ratio log2=1,471, q-value <0.0001), ACD (odds ratio log2=1,468 q-value=0.032), and TP53 (odds ratio log2=0.511 q -value =0.018) in TCGA cancers. Next, we analyzed the *TAPR1* gene expression and found that all tumors express this gene (data not shown).

Considering that *TAPR1* modulates p53, we compared the mRNA levels of this gene in relation of *TP53* mutation status (when available) (Figure 1B). Taking into account only the results with statistical significance (*p*<0.05), we found that *TAPR1* is more expressed in tumors (HNSC, LIHC, LUAD, PRAD, READ, and SKCM) with p53 mutations. This reinforces the oncogenic role of *TAPR1* when there are deficiencies in p53. The only exception to this phenomenon is in BRCA, in which *TAPR1* is upregulated in cancers without mutations in p53.

Next, we analyzed whether *TAPR1* has differential expression in cancer in relation to normal tissue (only in tumors where control is available) (Figure 2A). The UALCAN software indicated that *TAPR1* is significantly upregulated only in CHOL and HNSC. Finally, we analyzed whether *TAPR1* expression can predict cancer survival. First, we considered cancer as a whole and grouped all the TCGA samples. An increase of *TAPR1* expression was associated with poor overall survival, but the result lacked statistical significance (Figure 2B, HR=1.1, *p*=0.07). On the other hand, *TAPR1* may have a protective role in cancer progression since its downregulation increases the risk of disease relapse (Figure 2C, HR=0.86, *p*<0.0001). In the original study, the authors suggest that *TAPR1* may have a double behavior in cancer: it can suppress both apoptosis/senescence (oncogenic role) and the tumorigenesis process itself (tumor suppressive role).

The previous analysis did not elucidate the general role of *TAPR1* in cancer, so we verified its association with each cancer individually using a hazard ratio heat map. The upregulation of *TAPR1* was significantly associated (*p*<0.05) with poor overall survival in LGG and THCA (Figure 2D) and with disease relapse in BLCA (Figure 2E). These findings suggest an oncogenic role of *TAPR1* in these cancers. On the other hand, in KIRC, the upregulation of *TAPR1* had a protective role in terms of both overall (Figure 2D) and disease-free survival (Figure 2E), suggesting that the specific function of this gene varies according to the type of cancer.

P53 is probably the most important tumor suppressor in the human genome. Among its various functions, it keeps telomere length in check, thus limiting cell proliferation (12). For this reason, p53 interactors plays a fundamental role in oncology and is studied worldwide (13).

When Benslimane et al. (7) discovered a new regulator of the p53-telomere length axis, we were immediately intrigued and sought to understand the role of *TAPR1* in cancer. Our analyses reinforce the hypothesis that this newly identified gene may have an important role in oncology. Using TCGA datasets, we showed an association between *TAPR1* expression and p53 mutation status in several cancers and that this gene is differentially expressed in CHOL and HNSC.

In addition, our analysis indicates an association between *TAPR1* expression and cancer survival in some tumors. However, this association can be both positive and negative. This suggests that the role of this molecule is context-dependent and needs to be studied further. Evidently, more robust studies are needed to understand the exact role of *TAPR1* in disease progression, but this gene may become an important target in oncology or a new player in cancer biology.

## Figures and Tables

**Figure 1:**
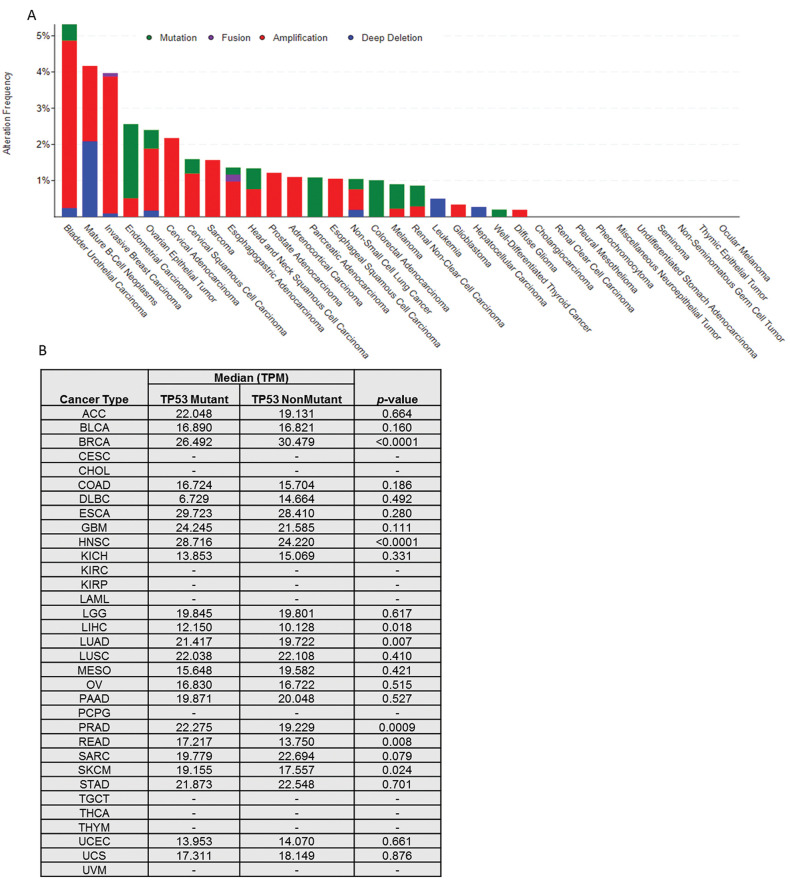
*TAPR1* in cancer. (A) Genomic alterations in each type of cancer and their proportions. (B) *TAPR1* mRNA levels in relation with TP53 mutation status. TPM=transcripts per million.

**Figure 2:**
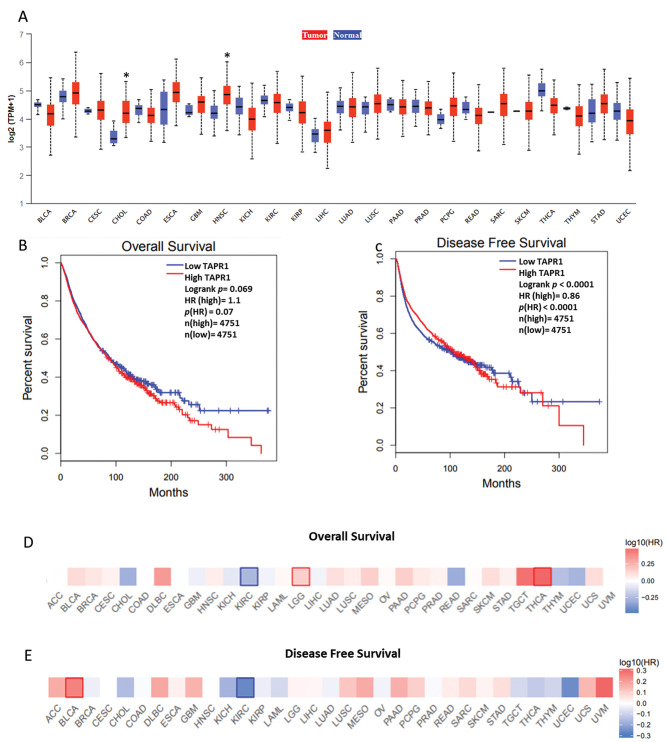
*TAPR1* and cancer prognosis. (A) Comparison of *TAPR1* expression between normal and cancer tissues. The boxplots are grouped in pairs for each cancer with the control tissue in blue (left) and the tumor tissue in red (right). (B) Overall survival and (C) disease-free survival considering all cancer samples. (D) Hazard ratio heat map of overall survival and (E) disease-free survival considering each cancer individually. Statistically significant results (Mantel-Cox test) are highlighted with blue (downregulated) or red (upregulated) borders. *p<0.05; HR=hazard ratio
